# Characterization of Feruloyl Esterase from *Klebsiella oxytoca* Z28 and Its Application in the Release of Ferulic Acid from De-Starching Wheat Bran

**DOI:** 10.3390/microorganisms11040989

**Published:** 2023-04-10

**Authors:** Yao Zhang, Zhiping Feng, Hongzhu Xiang, Xian Zhang, Lijuan Yang

**Affiliations:** 1 College of Bioengineering, Sichuan University of Science & Engineering, Yibin 644000, China; 2 Liquor Making Bio-Technology & Application of Key Laboratory of Sichuan Province, Sichuan University of Science & Engineering, Yibin 644000, China

**Keywords:** *Klebsiella oxytoca* Z28, feruloyl esterase, ferulic acid, de-starching wheat bran, biochemical characterization

## Abstract

Feruloyl esterase (EC3.1.1.73; FAE) can degrade biomass to release ferulic acid (FA), which has a high application in bioprocessing, food, pharmaceutical, paper, feed, and other industrial fields. A strain of *Klebsiella oxytoca* Z28 with ferulic esterase activity was screened from Daqu. In addition, the FAE gene was expressed in *Escherichia coli* BL21 (DE3). The enzyme consists of 340 amino acids with a molecular mass of 37.7 kDa. The FAE enzyme activity was 463 U/L when the substrate was ethyl 4-hydroxy-3-methoxycinnamate and the optimum temperature and pH were 50 °C and 8.0, respectively. The enzyme had good stability at temperatures of 25–40 °C and a pH of 8.0. Ba^2+^, Cu^2+^, Mn^2+^, and Ca^2+^ had a strong inhibitory effect on the enzyme activity, and Na^+^ had a promotive effect on the enzyme activity. The de-starching wheat bran was degraded by KoFAE, and the FA release was up to 227.15 µg/g. This indicated that the heterologous expression of KoFAE from *Klebsiella oxytoca* Z28 in *E. coli* had a certain potential of biodegradation, which can be applied to the degradation of agricultural waste to obtain high value-added FA products.

## 1. Introduction

Feruloyl esterase (E.C. 3.1.1.73, FAE), also known as cinnamic acid esterase, is a subclass of carboxylate esterase that hydrolyzes the ester bond break between ferulic acid (FA, 4-hydroxy-3-methoxycinnamic acid) and arabinoxylan or pectin, releasing FA monomer or dimer [1,2,3]. FAE is widely used in the feed, paper, and food industries. The addition of FAE in the feed processing process can accelerate the degradation of plant cell wall components, which is conducive to the digestion and absorption of animals, thereby promoting the growth and development of animals and improving the production performance of animals. [4]. Record et al. found that FAE, together with laccase and xylanase, has the effect of delignifying lignin when degrading wheatgrass pulp [5]. In the food industry, FAE can release FA present in agricultural waste such as wheat bran, beer pomace, and banana pomace [6]. FA is a strong antioxidant, and FA can be used to treat diabetes, cancer, cardiovascular and cerebrovascular diseases, and is widely used in health care products, food additives, cosmetics, and medicines [7,8,9].

Given the great potential of FAE for industrial applications, many FAE-producing microorganisms have been identified. Currently, FAE is mainly isolated from fungi such as *Aspergillus niger* [10], *Aspergillus oryzae* [11], *Aspergillus terreus* [12], and other fungi. Only a few studies have detected FAE in bacteria such as *Lactobacillus fermentum* [13], *Lactobacillus crispatus* [14], *Bacillus pumilus* [15], and *Clostridium thermocellum* [16]. The FAE derived from *Aspergillus niger* has been studied more intensively, and the FAE enzymatic activity of different sources varies greatly. Although the fungal FAE enzyme activity is high, its poor stability makes it difficult to meet large-scale production applications [17]. Relatively few studies have been conducted on FAEs from bacteria, such as *Butteriviribrio proteolaticus* [18], *Bacillus pumilus* [15]), and *Lactobacillus* [19]. Bacteria can be found in cold seas and in a variety of other environments, and enzymes produced by bacteria that are thermophilic or tolerant to extreme environments have superior biochemical properties [20]. Therefore, it is necessary to screen new bacteria with higher FAE activity, high enzyme activity, and good stability of FAE, so as to improve ferulic acid production from agricultural waste degradation.

Bacteria FAE produced by bacteria has been reported to have good FAE under acidic conditions. However, less has been reported about alkaline FAE. In this study, the Z28 strain with the highest FAE enzyme activity was screened from 37 strains in Daqu wine and distiller’s grains that had a degrading effect on ethyl 4-hydroxy-3-methoxycinnamate and identified as *Klebsiella oxytoca* Z28. There have been few reports of *Klebsiella oxytoca* FAE. Through sequence analysis, the potential FAE gene in the genome of *Klebsiella oxytoca* bacteria was discovered, and the expression of the gene in *E.coli* was realized. This enzyme has good FAE activity under alkaline conditions, and the enzyme was used to degrade starch-free wheat bran to release FA in order to lay a foundation for the combined release of FAE and lignocellulase to achieve factory production.

## 2. Materials and Methods

### 2.1. Materials and Reagents

Daqu liquor and distiller’s grain were collected from a distillery in Yibin, China; peptone, yeast extract, NaCl, (NH_4_)_2_SO_4_, KH_2_PO_4_, MgSO_4_·7H_2_O, CaCl_2_·H_2_O, and FeCl_3_ were purchased from Kelong Chemical Co., Ltd. (Chengdu, China); ferulic acid and ethyl 4-hydroxy-3-methoxycinnamate were purchased from Yuanye Biotechnology Co. Ltd. (Shanghai, China).

### 2.2. Culture Medium Conditions

The medium used for enrichment of microorganisms was an LB medium; the composition of the medium used for preliminary screening of microorganisms was (g/L): NaC1 0.3, ammonium sulfate 1.3, MgSO_4_·7H_2_O 0.3, K_2_HPO_4_ 0.3, agar 20, and 15 mL ethyl 4-hydroxy-3-methoxycinnamate, pH 7.0. Test tube liquid enzyme-producing medium (g/L): NaCl 10, yeast extract 5, peptone 10, and 5 mL ethyl 4-hydroxy-3-methoxycinnamate solution, pH 6.5. The composition of the screening medium used to assay FAE enzyme activity was (g/L): ammonium sulfate 1.3, KH_2_PO_4_ 0.37, MgSO_4_-7H_2_O 0.25, CaCl_2_-H_2_O 0.07, FeCl_3_ 0.02, yeast extract 5.0, and 5 mL ethyl 4-hydroxy-3-methoxycinnamate, pH 6.5. All media were sterilized at 121 °C for 20 min.

### 2.3. Screening of FAE Producing Strains

A total of 10 g of Daqu and distiller’s grain were weighed and added to a 250 mL conical flask. Each of them was added to a 250 mL conical flask with 90 mL of physiological saline and shaken in the conical flask for 30 min. The suspension was diluted to a certain number of times and then applied to the medium containing ethyl 4-hydroxy-3-methoxycinnamate and incubated in a constant temperature incubator at 37 °C for 72 h to observe the degradation of ethyl 4-hydroxy-3-methoxycinnamate by the strain. The strains with a larger circle size were selected for isolation and purification 3 times and then stored at −80 °C for storage. The seed solution was obtained by inoculating the preserved strain in the LB liquid medium for activation. The preserved strains were inoculated in the LB liquid medium and cultured in a shaker at 37 °C and 180 rpm for 24 h to obtain seed solution. The 2% (*v*/*v*) seed solution was inoculated by an in vitro enzyme-producing medium and cultured in a shaker at 37 °C and 180 rpm for 48 h. A total of 200 µL of the fermentation broth was placed in the Oxford cup added to the initial screening medium, and the medium was incubated at 37 °C for 48 h. The ability of FAE in the fermentation broth to decompose ethyl 4-hydroxy-3-methoxycinnamate was observed. The capacity of FAE production and FAE activity were preliminarily determined by the size of the hyaline ring.

According to the results of the clear circle test, the strain with the large clear circle produced by enzymatic hydrolysis was selected. They were seeded into the screening medium for FAE enzyme activity and incubated at 180 rpm, 37 °C for 72 h. The supernatant obtained by centrifugation of 50 mL fermentation broth at 7000 r/min for 10 min was the crude enzyme solution. The enzyme activity of FAE was determined by the decomposition of 4-hydroxy-3-methoxycinnamate ethyl ester as a substrate into FA to reflect its enzyme activity. In total, 1 mL crude enzyme solution was mixed with 2 mL 100 mM Tris HCl (pH 9.0) buffer solution containing 1% ethyl 4-hydroxy-3-methoxycinnamate. After allowing the enzyme to react with the substrate for 2 h at 37 °C, the reaction was terminated in an ice bath for 10 min. Ferulic acid production was measured at 320 nm by ultraviolet spectrophotometer. The above methods were compared with substrates without enzymes.

### 2.4. Identification and Phylogenetic Tree Analysis of Ferulic Esterase Producing Strains

FAE-producing strains were inoculated in the LB medium for 12 h after incubation at 37 °C, and colony morphology and a Gram stain were observed. The genome of the strain was extracted using the TIANamp Bacterial DNA Genome Extraction Kit. PCR amplification of the 16S rRNA gene was then performed, using universal primers of 27-F (5′-CAGAGTTTGATCCTGGCT-3′) and 1492-R (5′-AGGAGGTGATCCAGCCGCCGCA-3′). The PCR products were detected by 1% agarose gel electrophoresis and sent to Chengdu Qingke Company for sequencing. Sequencing results were compared with microorganisms in the GenBank database using the Blast created by the NCBI. Highly similar strain sequences were selected, and a phylogenetic tree was constructed using the neighbor-joining method (500 bootstrap copies) of MEGA 7 software.

### 2.5. Construction and Induced Expression of Recombinant FAEplasmid

The KoFae gene (Genbank accession number: WP_105889557) was amplified by PCR using *Klebsiella oxytoca* Z28 genomic DNA as a template, and the upstream and downstream primers (KoFae-F: 5′CGC GGATCC ATGAAAAAACTCTCCCTCCTG-3′; KoFae-R: 5′CCG CTCGAG TTACAGATTGGCGTTTTTATCT-3′). The PCR products were purified to obtain more pure DNA fragments. The purified DNA fragment was digested with BamHI and XhoI, and then the digested DNA fragment was connected to pET-28a to construct the recombinant plasmid pET-28a-KoFae. It was transformed into E.coli DH5α, and the plasmid was extracted and identified by double enzyme digestion. Plasmids identified as positive were sent to Qingke Biological Company for sequencing. The sequencing results of the sequence can be seen in the supplementary materials. The recombinant expression plasmid pET-28a-KoFae was transformed into *E. coli* BL21 (DE3), and the recombinant E.coli BL21/pET-28a-KoFae was cultured overnight and activated. The cultured seed solution was inoculated with LB medium containing resistance (50 μg/mL kanamycin) at 2% volume. It was incubated at 37 °C, 180 r/min for 2–3 h and then added 1 mM isopropyl beta-d-thiogalactoside (IPTG). It was then incubated at 25 °C, 180 r/min, for 8 h. Recombinant *E. coli* BL21/pET-28a-KoFae was collected by centrifugation at 4 °C and 7000 rpm. The cells were washed twice with 20 mm PBS (pH 7.4), resuspended, and ultrasonically broken. After centrifugation at 4 °C and 7000 rpm for 30 min, the supernatant was collected as a KoFAE enzyme crude solution.

### 2.6. Effects of Temperature and pH on Activity and Stability of FAE

The crude enzyme solution of KoFAE was reacted with the substrate ethyl 4-hydroxy-3-methoxycinnamate at the temperature of 25–60 °C to determine the optimum reaction temperature of the enzyme. The crude enzyme solution induced by *E. coli* containing pET-28a for the same time was used as a blank control. The residual enzyme activity was detected after the crude enzyme solution was kept at different temperatures for 1 h. The initial enzyme activity of the enzyme at different temperatures was 100% to determine the effect of temperature on the stability of the enzyme. Citrate–sodium citrate buffer (pH 3.0–5.0), phosphate buffer (pH 6.0–8.0), and glycine–sodium hydroxide buffer (pH 9.0–10.0) were prepared. The activity of FAE was measured under different pH conditions to determine the optimum pH of the enzyme. The residual enzyme activity of the crude enzyme solution was detected after it was stored at different pH for 1 h. The initial enzyme activity at different pH values was 100% to determine the effect of pH on enzyme stability.

### 2.7. Effects of Metal Ions on FAE Activity

The obtained crude enzyme solution was mixed with a final concentration of 1 mM metal ions (Co^2+^(CoCl_2_), Ba^2+^(BaCl_2_), Na^+^(NaCl), Cu^2+^(CuSO_4_), Zn^2+^(ZnSO_4_), Fe^3+^(FeSO_4_), Li^+^(LiCl), Mn^2+^(MnCl_2_), Mg^2+^(MgSO_4_), and Ca^2+^(CaCl_2_)) buffer solution. The crude enzyme solution induced by *E. coli* containing pET-28a for the same time was used as a blank control. After storage at 4 °C for 30 min, the enzyme activity was measured to determine the effects of metal ions on the enzyme activity.

### 2.8. Release of Ferulic Acid from KoFAE Hydrolyzed De-Amylated Wheat Bran

Deamylated bran was prepared as follows: 100 g of bran was mixed with an appropriate amount of water, boiled in a boiling water bath for 20 min to melt the starch, rinsed repeatedly with distilled water, and incubated in a 0.5% (*w*/*v*) amylase solution at 65 °C for 1 h with frequent stirring. Then, the solution was boiled at 99 °C for 30 min to inactivate the enzyme. Finally, the bran was repeatedly washed with distilled water to completely remove the starch. The obtained de-starch bran was dried to a constant weight. FA was produced from de-starching wheat bran by KoFAE. A total of 0.5 g wheat bran was suspended in 30 mL of 100 mM phosphate hydrochloric acid buffer (pH 8.0). In total, 10 mL KoFAE enzyme solution was added to the reaction system and incubated for 10 h at 150 r/min at 50 °C. After incubation, the reaction was terminated by boiling for 30 min and centrifuged at 8000× *g* for 10 min. The supernatant was collected and filtered for LC-MS analysis. LC-MS conditions: Waters Acquity UPLC Beh C18 1.7 μm 2.1 × 50 mm; eluent: 10% 0.1% acetic acid water, 90% methanol, column temperature 25 °C, flow rate 0.2 mL/min; mass spectrometry conditions: TEM 500 °C, IS-4500v, CUR 25psi, GS1 50 psi, GS2 50 psi, detection mode MRM, FA 192.9 > 134, DP-43v, EP-11V, CE-16V, CXP-5V.

## 3. Results and Discussion

### 3.1. Screening of Ferulic Esterase Producing Strains

**Figure 1:**
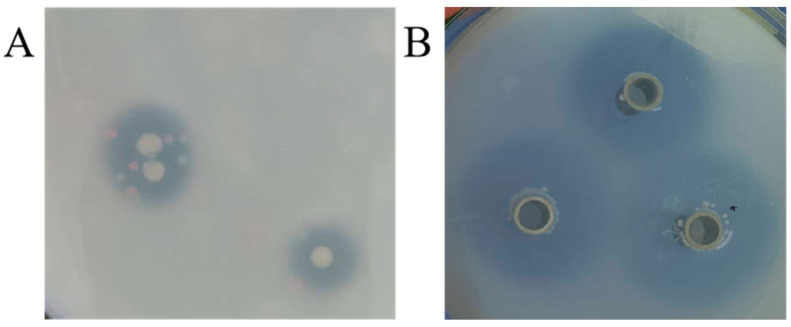
Clear circles produced by degradation of ethyl 4-hydroxy-3-methoxycinnamateas by the FAE strain (**A**), and clear circles produced by degradation of ethyl 4-hydroxy-3-methoxycinnamateas by the crude enzyme solution of FAE produced by strain (**B**).

Microorganisms enriched in Daqu were inoculated onto plates containing ethyl 4-hydroxy-3-methoxycinnamate. In this isolation and screening experiment, transparent circles appeared around some colonies (Figure 1A). This is because strains with FAE activity break down ethyl 4-hydroxy-3-methoxycinnamate to form clear circles. The above strains were incubated in liquid medium for 48 h, and the FAE activity was evaluated by Oxford cup clear circle assay (Figure 1B). The decomposition of ethyl 4-hydroxy-3-methoxycinnamate in the medium from the crude enzyme solution of 37 screened strains was shown in Table 1. Compared with the size of the Oxford cup clear circle, the Z28 strain had the largest transparent circle diameter, and the FAE enzyme activity of the strains were also measured, with the Z28 strain having the highest enzyme activity of 35.00 U/L (Table 2).

### 3.2. Identification of Ferulic Esterase Producing Strain

**Figure 2:**
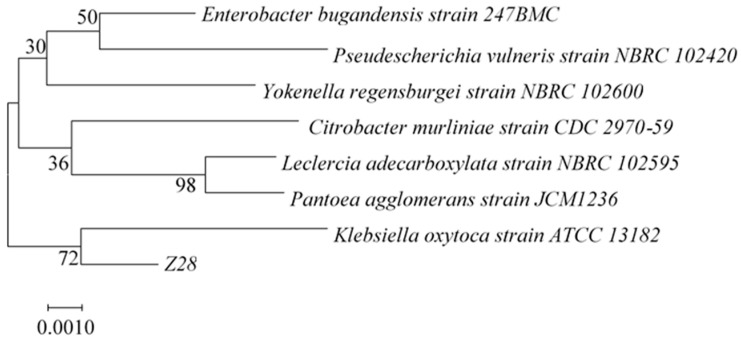
Identification of strains Z28 by 16 S rRNA sequencing after constructing the phylogenetic tree using the neighbor-joining method at 500 replicates of bootstrap value.

The Z28 strain was cultured on an LB medium to observe the morphology of single colonies (Figure S1A), and it was found that it was a round colony with a smooth surface, grayish white, and sticky silk when lifted. The Z28 strain showed a red color by Gram staining under the microscope, as shown in Figure S1B, and was judged to be a Gram-negative bacteria. The 16S rRNA gene sequence of Z28 has 99.5% homology with *Klebsiella oxytoca*. To determine the genetic relationship of the target strain, the phylogenetic tree was constructed by the neighbor-joining method of MEGA 7 software, as shown in Figure 2. The genetic distance between the Z28 strain and *Klebsiella oxytoca* is the closest, so it was named *Klebsiella oxytoca* Z28.

### 3.3. Cloning and Expression of KoFae Gene

**Figure 3:**
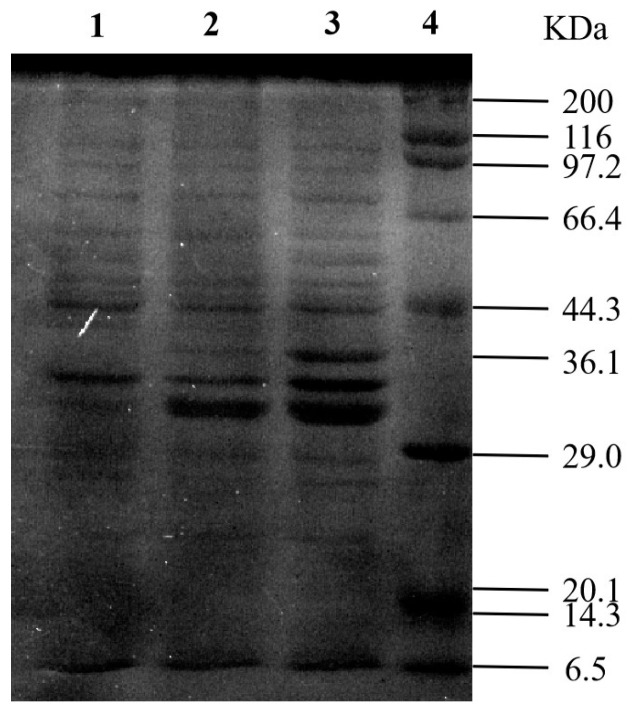
Analysis of feruloyl esterase (KoFae) by SDS-PAGE. Lane 1: IPTG-induced crude protein expressed by *E. coli* carrying plasmid pET-28a; lane 2: IPTG-induced ultrasound-treated supernatant of *E. coli* carrying plasmid pET-28a-KoFae; lane 3: I IPTG-induced crude protein expressed by *E. coli* carrying plasmid pET-28a-KoFae; lane 4: standard protein marker.

Based on the analysis of gene sequences of FAEs from different sources, we speculated about the sequence of the ferulate-esterase gene of *Klebsiella oxytoca*, and the full-length nucleotide sequence of the gene (1023 bp) has been submitted to the GenBank nucleotide sequence database (registration number: WP_105889557). The KoFae gene was amplified by PCR using the *Klebsiella oxytoca* Z28 genome as the template, and the characteristic band appeared at about 1023 bp. The PCR product was ligated onto the pET-28a vector, and the target band of about 1023 bp was identified by double enzyme digestion. The sequencing results were consistent with the results published by GenBank. The KoFae open reading frame was 1023 bp, encoding 341 amino acid residues. The molecular mass and pI were 36,127.33 kDa and 7.81, respectively (https://web.expasy.org/protparam/, accessed on 18 November 2022). The protein is named KoFAE. The grand average of the hydropathicity of koFAE was −0.049, indicative of a hydrophilic protein. By comparing KoFAE with FAE protein sequences previously reported, the results showed that the homology of Kofae with FAE derived from *Klebsiella pneumoniae* (GenBank ID: BCI97608.1) was the highest 40.74%. The homology between KoFAE and FAE with *Lactobacillus crispatus* (GenBank ID: MK640209.1) and *B. altitudinis* (GenBank ID: AKC64980) was 39.58% and 31.31%, respectively. In addition, it was found that a “Gly-X-Ser-X-Gly” motif was observed in the sequence, which was highly conserved in previously reported FAE. *E.coli* BL21(DE3) was transformed with the pET-28a-koFae plasmid, and after 0.5 mmol/L IPTG induced expression, SDS-PAGE electrophoresis detected the target band at about 36.13 kDa (Figure 3). *E.coli* BL21(DE3) containing pET-28a and pET-28a-KoFae were inoculated onto plates containing ethyl 4-hydroxy-3-methoxycinnamate (Figure S2), and transparent circles were observed on plates containing pET-28a-KoFae, which had FAE activity and could decompose ethyl 4-hydroxy-3-methoxycinnamate. FAE activity could be reached at 463 U/L. The heterologous expression of FAE from *Klebsiella oxytoca* Z28 in *E. coli* was first completed.

### 3.4. Influence of Temperature and pH on Activity and Stability of FAE

**Figure 4:**
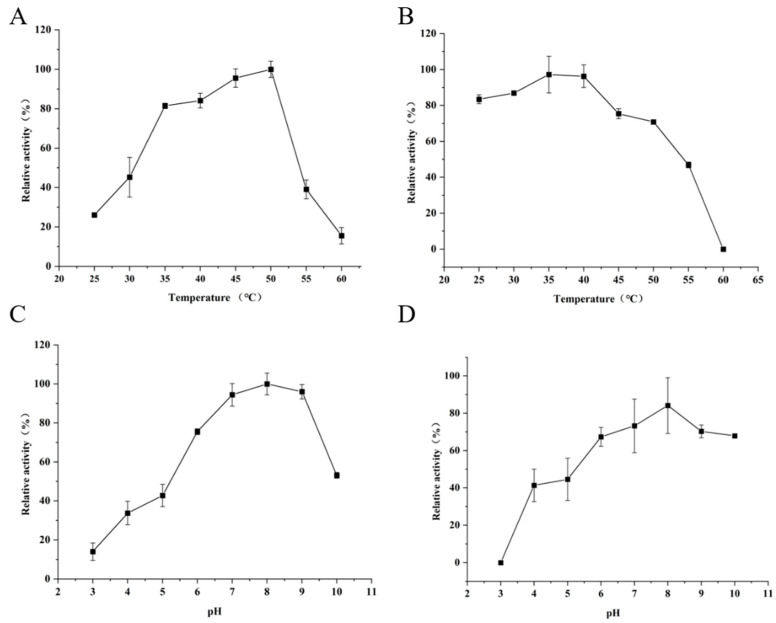
Determination of enzymatic properties of Feruloyl esterase (KoFAE). (**A**) Effect of different temperatures on enzymatic activity of KoFAE. (**B**) The temperature stability of KoFAE. (**C**) Effect of different pH on enzymatic activity of KoFAE. (**D**) The pH stability of KoFAE. The data were acquired in triplicate experiments.

The optimal temperature and temperature stability of KoFAE were evaluated (Figure 4A,B). When the temperature was 25–60 °C, the enzyme activity of FAE increased first and then decreased with the increase of temperature. When the temperature was 50 °C, the enzyme activity reached the highest. When the temperature was higher than 45 °C, the stability of the enzyme activity decreased sharply. Most studies found that the optimal reaction temperature of FAE was closely related to the FAE-producing strain, such as *Bacteroides intestinalis* (37 °C) [21], *Penicillium piceum* (70 °C) [22], and *Lactarius hatsudake* (30 °C) [23]. Xu et al. [14] found that FAE from *Lactobacillus* has good thermal stability at the temperature of 25~50 °C. KoFAE in this study had good thermal stability when the temperature ranged from 25 °C to 40 °C. When the temperature increased from 45 °C to 50 °C, the stability decreased slowly, and more than 60% of the enzyme activity still remained. The optimal reaction pH for KoFAE is shown in Figure 4C. Under the acidic condition of pH 3.0, the enzyme had almost no activity. When pH was 4.0–7.0, the enzyme activity increased with the increase of pH. When pH = 8.0, the enzyme activity reached the highest. When pH > 8.0, the enzyme activity began to decrease, but about 90% of the enzyme activity was retained when pH = 9.0. When pH > 9.0, the enzyme activity decreased sharply. When pH 10.0, less than 50% of the enzyme activity was retained. As for the pH stability of KoFAE, it can be seen in Figure 4D that the enzyme activity was less stable when pH was 3.0 to 5.0, the enzyme activity was lost when pH was 3.0, and the stability was less than 50% when pH was 4.0 to 5.0. A pH of 6.0~10.0 had good stability, the retained enzyme activity was more than 60%, and pH 8.0, the best stability of enzyme activity, could retain about 80% of enzyme activity. Shu et al. screened the FAE activity gene estF27 from the soil metagenic library, which also showed good activity at pH 8, and its stability could preserve 80% of the enzyme activity at pH 8 [24]. Zhang et al. studied Bi76, a protein with FAE activity from *B. intestinalis*. It has good activity and stability at pH 5.5, but the FAE enzyme activity is less than 10% at pH 8.0 [25]. This result indicates that KoFAE is a relatively stable protein under alkaline conditions.

### 3.5. Effect of Metal Ions on KoFAE Activity

The effects of metal ions on the enzymatic activity of KoFAE were measured (Table 3). The results showed that most metal ions inhibited the activity of KoFAE enzyme in different degrees, and Ba^2+^, Cu^2+^, Mn^2+^, and Ca^+^ inhibited the activity of the Kofae enzyme by more than 40%. However, Co^+^, Zn^2+^, Fe^3+^, and Mg^2+^ inhibited the enzyme activity by about 20%, while Na^+^ had a slight promotion effect on the enzyme activity. Different from previous studies, Agrawal et al. found that Mg^2+^, Ca^+^, and Cu^2+^ promoted FAE derived from *Pichia pastoris* X33, while Na^+^ inhibited FAE [26]. This reflects that sodium ions play different roles on FAEs from different sources—some inhibit and some promote, reflecting the species diversity of FAEs.

### 3.6. KoFAE Hydrolyzes de-Amylated Wheat Bran to Release FA

An important current application of FAE is the degradation of lignocellulose to release FA [27]. De-starched wheat bran was used as the substrate and added to KoFAE to hydrolyze the de-starched wheat bran for 10 h. The FA yield was determined by the LC-MS method, and the crude enzyme solution obtained by plasmid transformation without the target gene was used as a blank control. The results showed that the yield of FA was not detected in the blank sample, and the FA yield from 1.0 g de-starched wheat bran was 227.15 μg after ferulate esterase degradation for 10 h. Liangkun et al. [28] obtained 4.04 mg/g FA from de-amylated wheat bran, and the FA released by enzymatic reaction accounted for 70% of the total FA. Xiaoli et al. [15] found that only 5% and 2% FA was released from de-amylated wheat bran when purified FAE and commercial xylanase were used alone, but in the presence of FAE and xylanase, the FA released was approximately 10-fold compared to FAE alone. Xu et al. [14] used *Lactobacillus*-derived FAE to release up to 199 μg FA from 0.2 g de-amylated wheat bran. In this study, FA can be obtained in a short time by using FAE derived from *Klebsiella oxytoca*, which promotes the development of the research on the release of FA from the degradation of lignocellulosic waste. According to the characteristics of this enzyme, which belongs to alkaline FAE, subsequent studies can study its ability to release FAE from waste under alkaline conditions, so as to meet the actual industrial demand.

## 4. Conclusions

In this study, a strain of *Klebsiella oxytoca* Z28 with FAE activity was obtained from the liquor Daqu, and its FAE activity reached 35.0 U/L. In addition, for the first time, an alkaline FAE was identified from *Klebsiella oxytoca* and heterologous expressed in *Escherichia coli*. The enzyme activity of the crude KoFAE enzyme solution reached 463 U/L when ethyl 4-hydroxy-3-methoxycinnamate was used as substrate. The enzyme was stable at 25~40 °C and pH 8.0, and Na^+^ could activate the activity of KoFAE. Using the crude KoFAE enzyme solution directly on de-amylated wheat bran, the FA release could reach 227.15 µg/g. This work enriched the resources of ferulic acid-releasing strains, provided an FAE derived from *Klebsiella oxytoca,* and provided an experimental basis for bioextraction of FA from agricultural waste species.

## Figures and Tables

**Table 1:** Clear circles produced by the FAE producing strain.

Strain No.	Diameter of t Clear Circles (mm)	Strain No.	Diameter of Clear Circles (mm)
Z6	22.33	1-3	15.00
Z28	22.87	2-1	0.00
J1	2.00	2-2	21.00
J2	0.00	2-3	2.67
J3	2.83	2-4	21.00
J4	0.67	3-1	14.00
D1	8.33	3-2	17.00
D2	6.07	3-3	19.00
D3	8.97	4-1	16.67
D4	12.83	4-2	15.33
D5	17.00	4-3	11.33
D8	7.67	5-1	7.00
D9	18.33	5-2	14.00
D6	21.33	5-3	22.67
D7	17.00	5-4	19.67
LC9	19.00	6-1	6.33
JW1	18.00	6-2	11.00
JW2	2.07		
1-1	17.67		
1-2	17.00		

Note: the data were acquired in triplicate experiments.

**Table 2:** FAE enzymatic assay of the strains.

Strain No.	FAE Activity (U/L)	Strain No.	FAE Activity (U/L)
D5	18.5 ± 0.5	1-2	23.4 ± 0.4
D6	25.2 ± 0.3	2-2	7.7 ± 0.3
D7	32.2 ± 0.5	2-4	3.1 ± 0.5
D9	16.0 ± 0.5	3-1	13.7 ± 0.5
Z6	18.7 ± 0.4	3-2	1.7 ± 0.1
Z28	35.0 ± 0.6	3-3	34.0 ± 0.3
J1	30.1 ± 0.6	4-2	7.9 ± 0.3
JW1	29.0 ± 0.3	5-3	4.5 ± 0.4
JW2	34.9 ± 0.1	5-4	14.9 ± 0.5
LC9	17.9 ± 0.1	6-1	8.8 ± 0.3

Note: The data are presented as the means ± standard deviations of three independent experiments.

**Table 3:** Effect of metal ions on KoFAE enzyme activity.

Metal Ions and Organic Compounds	Relative Activity/(%)
Co^2+^	78.17 ± 4.48
Ba^2+^	58.22 ± 6.15
Na^+^	105.95 ± 2.90
Cu^2+^	50.86 ± 5.23
Zn^2+^	81.14 ± 5.16
Fe^3+^	79.05 ± 4.08
Li^+^	72.85 ± 6.96
Mn^2+^	60.33 ± 6.73
Mg^2+^	79.42 ± 7.52
Ca^2+^	55.56 ± 5.21

Note: the data are presented as the means ± standard deviations of three independent experiments.

## Data Availability

Date is contained within the article.

## Supplementary Materials

The following supporting information can be downloaded at: https://www.mdpi.com/article/10.3390/microorganisms11040989/s1, Figure S1: Colony morphology of *Klebsiella oxytoca* Z28 in the medium(A) and Gram staining results of *Klebsiella oxytoca* Z28 under microscope(B); Figure S2:The control group was the degradation of ethyl 4-hydroxy-3-methoxycinnamate in LB medium by Escherichia coli BL (DE3) containing pET-28a (A); experimental group: degradation of ethyl 4-hydroxy-3-methoxycinnamate in LB medium by Escherichia coli BL (DE3) containing pER-28a-KoFae (B); Figure S3: The peak area of ferulic acid was determined by LC-MS; A: Standard sample; B: Sample 1: C: Sample 2; Table S1: The original data of ferulic acid yield detected by LC-MS.
