# Supplementary material for: Characterization of Feruloyl Esterase from Klebsiella oxytoca Z28 and Its Application in the Release of Ferulic Acid from De-Starching Wheat Bran
Source: Microorganisms. 2023 Apr 10;11(4):989. doi: 10.3390/microorganisms11040989 (PMC10146132; doi:10.3390/microorganisms11040989)
Supplement: Supplementary file 1 [file microorganisms-11-00989-s001.zip › microorganisms-2321903-supplementary.pdf]

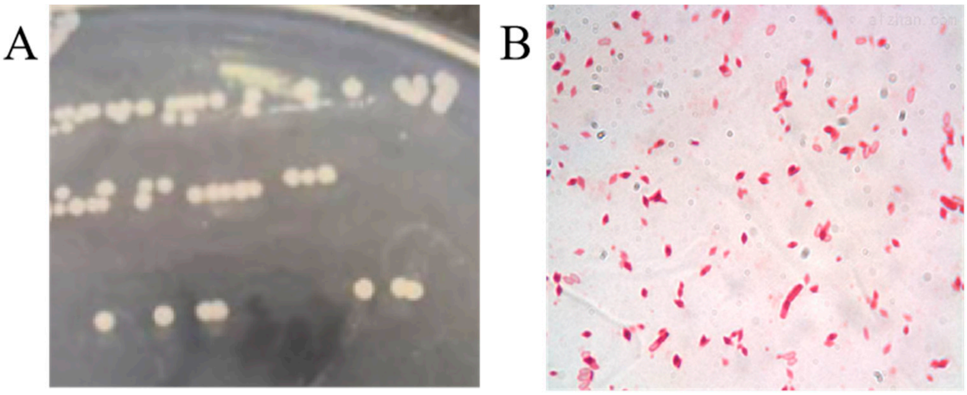

Figure S1. Colony morphology of *Klebsiella oxytoca* Z28 in the medium(A) and Gram staining results of *Klebsiella oxytoca* Z28 under microscope(B).

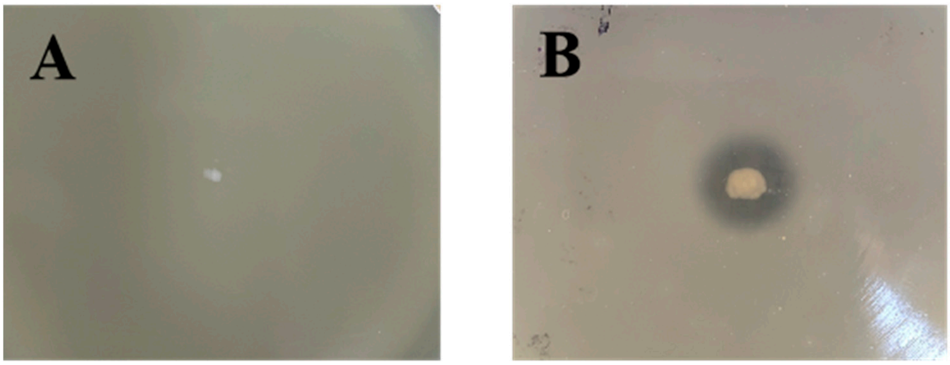

Figure S2. The control group was the degradation of ethyl 4-hydroxy-3-methoxycinnamate in LB medium by *Escherichia coli* BL（DE3）containing pET-28a (A); experimental group: degradation of ethyl 4-hydroxy-3-methoxycinnamate in LB medium by *Escherichia coli* BL（DE3）containing pER-28a-KoFae (B).

The gene sequence of koFAE：ATGAAAAAACTCTCCCTCCTGCTCACCGGCGGCCTGCT
GCTTGCCGGCATGGCTTCCTGCGTTAACGCCGGCGAACTTGCTGGCAATACCGAAGGC
GCTCAGGTCATTCGCGTGGAAAAAACCCGGGGCCGTATCGATGCGCTCAACGATATCG
TTTATAGCCAGGTAAAAAGCACCGTCGCCGTTCGTCAGCTGCATATGTCTTTACTGGTA
CCGCGTAACGGCGATCTGAAACCGGCAATCATCTATTTCCCCGGCGGCGGTTTCACCA
GCGCGGCCTGGAATAAGTTTATCGAGATGCGTATGGCGCTGGCGGCGGCGGGATTCGT
CGTCGCGGCGGCGGAATACCGTACGGTTCCCGACACCTTTCCGGCCCCGGTTATCGATG
GTAAAGCCGCCGTTCGCTATCTGCGCGAACATGCCGCGGAATACGGTATAGACCCGTCG
CGTATTGGCGTGCTGGGGGATTCCGCAGGCGGCTATATGGCGCAGATGCTGGCGCTCAC
CAACGGTGACAAAACGTTCGAGCGTGGCGATAACCTGGCACAATCCTCGGATGTGCAG
GCCGCCGCCACGCTGTACGGTATCTCGAACCTGTTGAATATCGGCGAAGGTTTTCCGCA
GGCGATTCAAAAAGTGCACCAGTCGCCGGCGGTCACTGAAGCGCTGCTGGTACACGG
TAGCGCCTTTCGCGACTGGCCGGGAGCGACGATTGACAGCGATCCGCAAAAAGCCCT
GGCGGCCAGCCCGATGGGCCATATCAGCGGTAAAAAGCCGCCGTTTTTAATTATGCACG

GCAGCGCCGACACCCTGGTTTCACCGGTACAAAGTCAACAGCTCTATCGCGCGCTGAA
AAAGGAGGGCAACCCGGCCGATTACGTGTTGCTGGCGGGCGCGGAGCACGGCGATGA
CAGCTGGTATCAGAAGCCGGTTATTGAACGGGTGGTGAACTGGTTTAAAGCGACCCTC
GGCGCGCCCAAAGCCGCCGCGACGGCAGAGAAAGATAAAAACGCCAATCTGTAA

Amino acid sequence of koFAE：MKKLSLLLTGGLLLAGMASCVNAGELAGNTEGAQVIR
VEKTRGRIDALNDIVYSQVKSTVAVRQLHMSLLVPRNGDLKPAIIYFPGGGFTSAAWNKFI
EMRMALAAAGFVVAAAEYRTVPDTFPAPVIDGKAAVRYLREHAAEYGIDPSRIGVLGDSA
GGYMAQMLALTNGDKTFERGDNLAQSSDVQAAATLYGISNLLNIGEGFPQAIQKVHQSPA
VTEALLVHGSAFRDWPGATIDSDPQKALAASPMGHISGKKPPFLIMHGSADTLVSPVQSQ
QLYRALKKEGNPADYVLLAGAEHGDDSWYQKPVIERVVNWFKATLGAPKAAATAEKDK
NANL

Table S1. The original data of ferulic acid yield detected by LC-MS.

sample name	Peak area	Ferulic acid yield（ug/g）	average（ug/g）
Standard sample（50ug/L）	15100000	-	-
Sample 1	32689386	237.82	227.15
Sample 2	35910578	216.48	

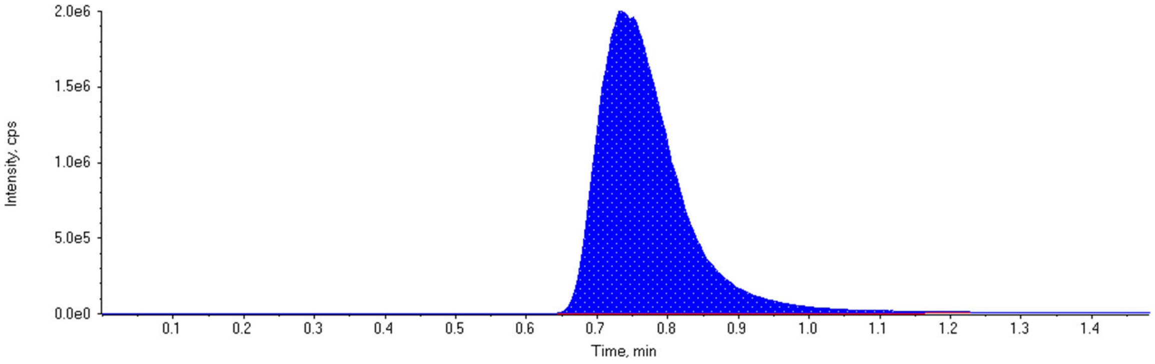

（A）

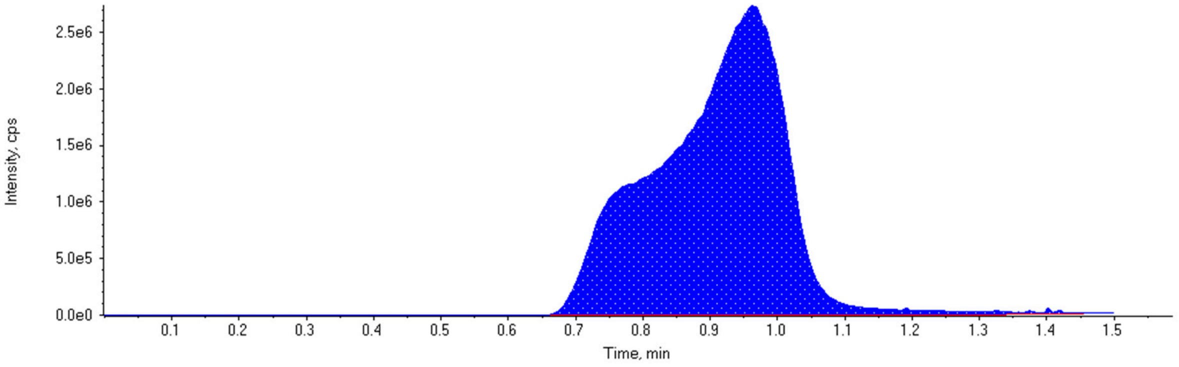

（B）

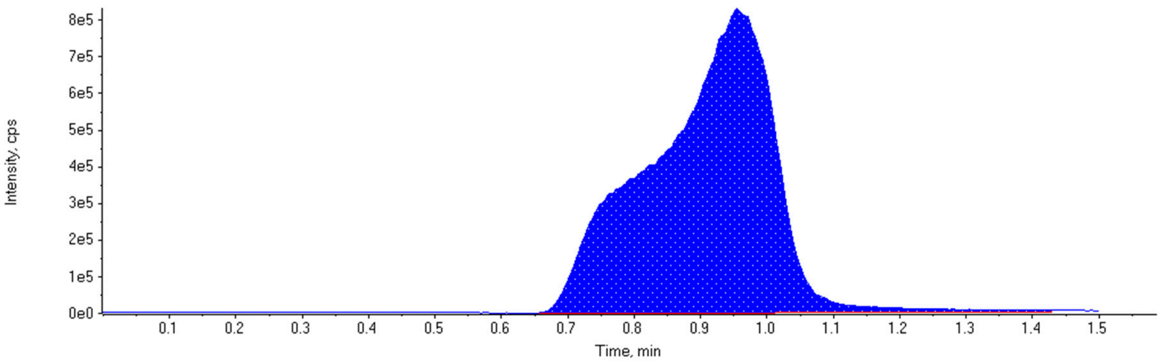

（C）

Figure S3. The peak area of ferulic acid was determined by LC-MS；A：Standard sample；B：Sample 1；C：Sample 2.
